# A Case of Adult-Onset IgA Vasculitis in a Cirrhotic Patient

**DOI:** 10.7759/cureus.27812

**Published:** 2022-08-09

**Authors:** Izza Bazigh, Etee Patel, Uzair Khan, Neha Ghalib, Aaparna Singh

**Affiliations:** 1 Internal Medicine, HCA Oak Hill Hospital, Spring Hill, USA

**Keywords:** iga nephropathy, alcoholic liver cirrhosis, liver cirrhosis, henoch-schönlein purpura, immunoglobulin a vasculitis

## Abstract

Immunoglobulin A vasculitis (IgAV; formerly called Henoch-Schönlein purpura) is a disease commonly seen in children as an immune reaction after a viral infection. It is a small vessel vasculitis characterized by immune complex deposits in various organs throughout the body. It mainly affects the skin, joints, abdomen and kidneys. This presentation is less likely to be seen in adults. In adults, IgAV can be seen due to decreased clearance of immune complexes through the liver. A damaged liver due to alcoholic liver cirrhosis can hinder the clearance of IgA complexes. We present an unusual case of a 42-year-old female who presented with alcoholic liver cirrhosis and ascites and later developed a purpuric rash in her lower extremities.

## Introduction

Immunoglobulin Ig vasculitis (IgAV) is a small-vessel vasculitis that affects multiple organs. Around 90% of cases occur in the pediatric population. Annual incidence of IgAV is estimated at 0.8-1.8/100,000 in adults and 3-26.7/100,000 in children [1]. The 1990 American College of Rheumatology (ACR) criteria for the diagnosis of IgAV include palpable pupura, age of onset < 20 years, acute abdominal pain, and biopsy showing granulocytes in the walls of small vessels. However, these criteria were appropriate only in the pediatric setting with striking features of IgAV and lacked diagnostic sensitivity and specificity in adults. More recently, in 2010, the European Alliance of Associations for Rheumatology (EULAR; formerly European League against Rheumatism), PRINTO (Paediatric Rheumatology International Trials Organisation), and PReS (Paediatric Rheumatology European Society) proposed a new consensus criteria for diagnosing IgAV that had higher sensitivity in adult population [2]. This included the mandatory presence of palpable purpura with lower extremities predominance with at least one of the following: diffuse abdominal pain, any tissue biopsy showing IgA deposition, arthritis or arthralgias, or renal involvement.

## Case presentation

**Figure 1:**
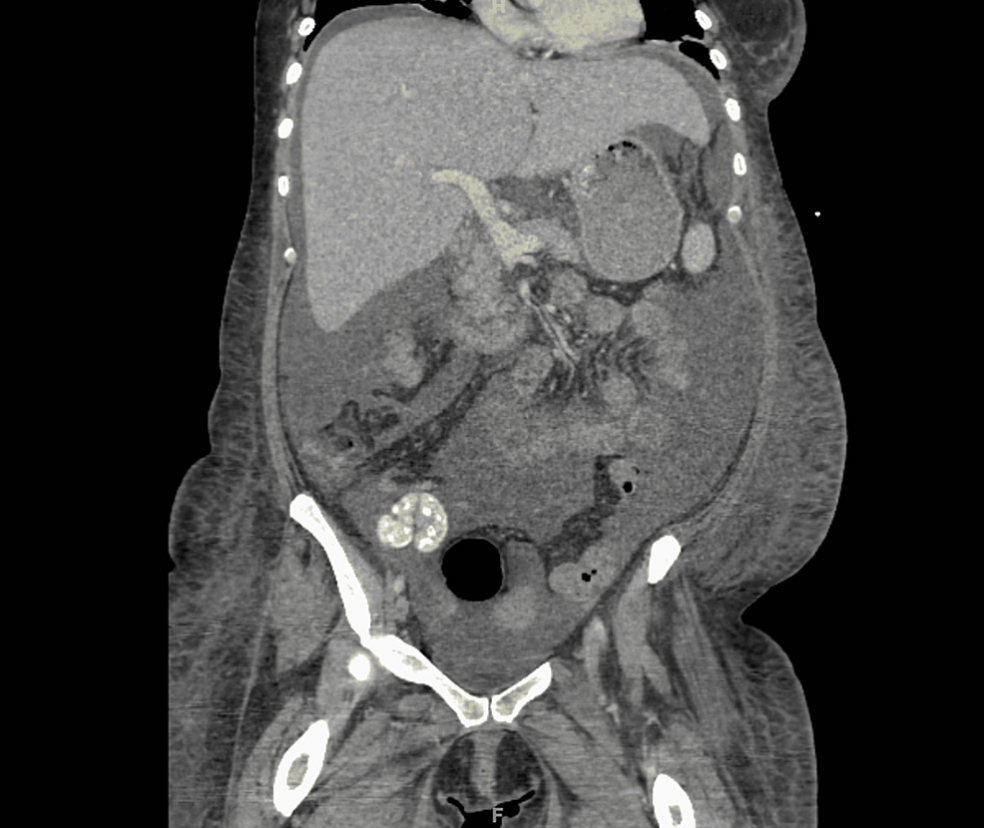
CT of the abdomen/pelvis with IV contrast (sagittal view)

**Figure 2:**
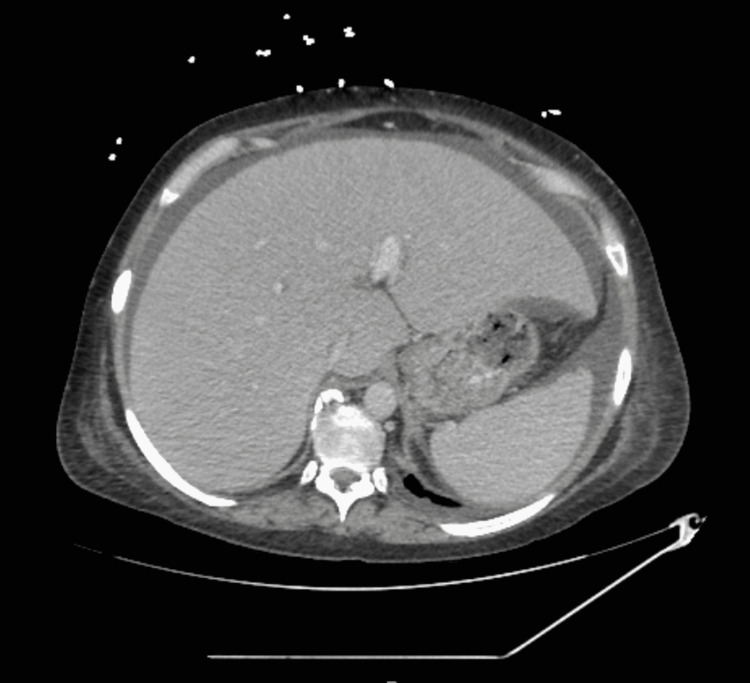
CT of the abdomen/pelvis with IV contrast (cross-sectional view)

Our patient is a 42-year-old female with a past medical history of liver cirrhosis, chronic pancreatitis, alcohol dependence, and hidradenitis suppurativa, who was hospitalized for abdominal pain. She was discharged from a local hospital two weeks prior for a similar presentation where a paracentesis was performed that partially alleviated her pain. On examination, the patient stated that her pain was diffuse, non-radiating, and 10/10 in intensity. She was tachycardic and had an elevated white blood cell count of 24.8 x 10^3^/uL. Her erythrocyte sedimentation rate (ESR) was elevated at 140 mm/hr, and C-reactive protein level was 6.3 mg/dL. Computerized tomography (CT) of the abdomen and pelvis with intravenous contrast revealed hepatosplenomegaly, large amount of ascites of uncertain etiology, recanalized umbilical vein suggesting portal hypertension, and diffuse subcutaneous edema (Figures 1, 2).

**Figure 3:**
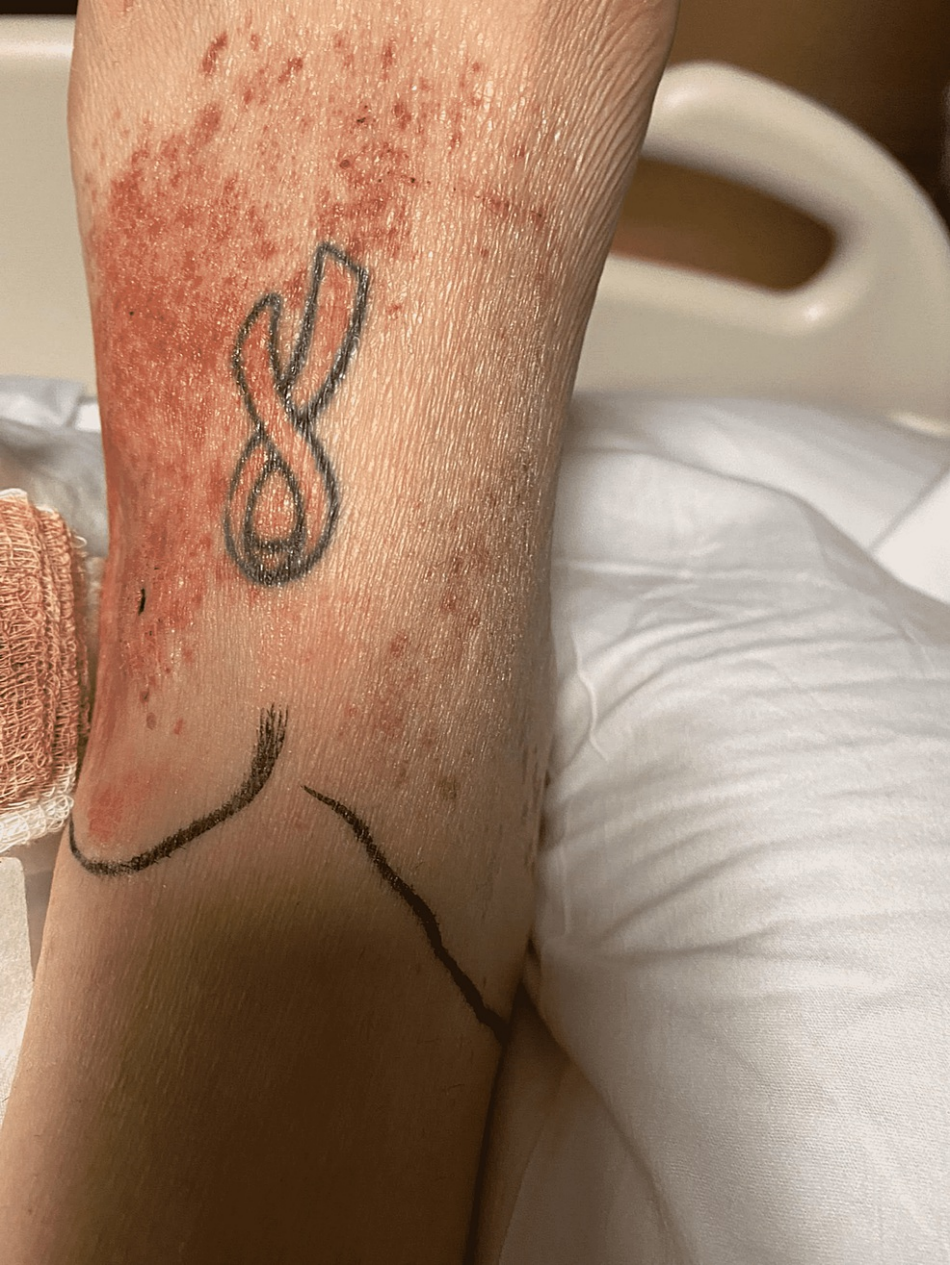
Vasculitic rash in lower extremities

Ultrasound-guided paracentesis was performed twice with a total of approximately 7 liters of fluid removed. Fluid analysis was consistent with transudative etiology and showed no evidence of spontaneous bacterial peritonitis (SBP). Her hepatitis panel was negative. Her autoimmune panel was negative for anti-nuclear antibodies (ANA), anti-neutrophil cytoplasm antibodies (ANCA), and rheumatoid factors (RF). Complement levels were low with C3 at 85 mg/dL and C4 at 11 mg/dL. Immunoglobulin screen showed that her serum IgA level was elevated at 589 mg/dL (normal range: <400 mg/dL). On day 5 of admission, the patient started complaining of a painful non-blanching purpuric rash on her lower extremities (Figure 3).

General surgery and rheumatology services were consulted for skin biopsy and evaluation of possible vasculitis. She was started on steroids. Her biopsy results were nonspecific because biopsy was taken after 24 hours of eruption of rash. It has been observed that the positivity of biopsy results declines after a few days of rash onset. Unfortunately, for our patient, punch biopsy of skin was delayed and performed on day 7, when positivity of biopsy drops to 15%. Her biopsy results showed superficial erythrocyte extravasation and sparse perivascular inflammation consisting of lymphocytes and histiocytes. Some of the expected biopsy findings include fibrinoid necrosis, inflammatory cells infiltrating the adventitia and media, and extravasation of red blood cells causing purpura. Multiple authors agree that a biopsy should be performed between 24 and 48 hours of lesion onset to increase the diagnostic yield [3]. Ideally, a representative lesion should be sampled when it is relatively “fresh” yet well-established [4].

Because our patient lacked systemic findings of large or medium vessel vasculitis, we narrowed our differential diagnosis to a cutaneous small vessel vasculitis. Her C-ANCA and P-ANCA were negative, which ruled out ANCA-associated vasculitis. Among the immune-mediated small vessel vasculitides, hypersensitivity vasculitis would be a strong consideration, but elevated IgA levels and the pattern of skin involvement below the ankles, as well as the biopsy negative for LCV (leukocytoclastic vasculitis) made us steer away from this diagnosis. Urticarial vasculitis would be recurrent and present with systemic involvement. Rheumatoid Arthritis associated vasculitis would be uncommon with negative serologies.

After extensive workup for possible causes of her purpuric rash, she was diagnosed with IgAV as per the ACR, EuLAR, and PReS criteria. The patient reported that she felt better and decided that she wanted to go home and follow-up as an outpatient. She was counseled on alcohol cessation and further complications arising from her cirrhotic liver.

## Discussion

IgAV, traditionally known as Henoch-Schönlein purpura (HSP), is the most common vasculitis seen in children. It presents as a rash that erupts more often in the fall and winter months and usually following a viral infection. The pathogenesis is not fully understood but it is postulated that IgA immune complexes (galactose-deficient (Gd-)IgA1) activate complement via IgA Fc receptor FcαRI (CD89), and the subsequent neutrophil migration leads to vascular inflammation [5]. An alternate theory is that antibodies against IgA (IgAV IgA1) are generated, which target endothelial cells [6].

In a case series report, among 20 patients with underlying cirrhosis, IgAV was a co-occurrence in 12 (60%) patients. Cirrhosis was mainly related to alcohol intake (n = 15, 75%) [7]. In alcoholic liver disease, there is an elevation in the serum IgA level, and this elevation gives rise to the formation of the IgA containing immune complexes (IgA-CIC), which, in turn, leads to this immune complex deposition in the tunica media of small vessels and offsets an inflammatory response. A study compared the IgA-CIC levels in 2 groups of patients with differing histopathological patterns of liver disease: 41 patients with alcoholic liver disease and 41 patients with non-alcoholic liver disease. It concluded that the presence and concentration of IgA-CIC is lowest in cases with nonspecific changes or steatosis seen on liver biopsy and highest in cases with hepatitis or cirrhosis (p-value less than 0.01). The study results implied that a direct correlation exists between the concentration of IgA-CIC and the severity of liver damage [5].

The mechanism whereby alcohol use leads to increase in IgA immune complex deposition is unclear, but several theories have been proposed. In the context of liver injury, alcohol induces the expression of the microsomal ethanol oxidation system (CYP2E1), and the oxidative stress resulting from increased production of reactive oxygen species leads to an excessive number of free radicals, which, in turn, trigger tissue injury and increase inflammation [8]. IgAV has been observed in a case with alcoholic cirrhosis and SBP [9]. Similarly, it has also been seen in a patient with liver cirrhosis due to Wilson's disease [10].

Because IgAV is not just confined to the skin, it can also manifest in the kidneys, gastrointestinal (GI) tract, and joints. Cirrhotic glomerulonephritis is a type of secondary IgA nephropathy that occurs in alcoholic cirrhotic patients. Due to the defect in hepatic clearance on IgA and increased production of IgA, abnormal IgA deposits in the renal mesangium and leads to injury. Large IgA1 immune complexes are formed from the abnormal glycosylation of IgA1 combined with IgG and IgA autoantibodies [11]. In decompensated cirrhotic patients, the glycosylation is more evident. It is thus hypothesized that this increased IgA synthesis and decreased clearance by hepatic phagocytes could lead to cirrhotic glomerulonephritis, or secondary IgA nephropathy [12]. The relationship between excessive alcohol intake-associated hepatocellular injury and IgA nephropathy has been validated in rat experiments. Intense IgA deposition, mild mesangial expansion, and foot process effacement have been observed in these animal models following chronic ethanol ingestion [13]. IgA1 has a modified N-glycosylation that is found only in alcoholic cirrhosis, and these IgA deposits were linked with CD71 overexpression in the mesangium. Along with deposition in the kidneys, IgA-immune complexes accumulate in the small vessels of joints, skin, and the GI tract, which, in turn, activate neutrophils through the Fc receptor (CD89), leading to tissue damage and palpable purpura. In a 13-year-long observational study of a total cohort of 118 patients with liver cirrhosis, nine with acute proliferative glomerulonephritis (APGN) were seen to have unusually large size of IgA glomerular complexes; 67 others also showed IgA deposits, albeit small [14].

Upon review of the literature, it is apparent that while IgA nephropathy is well associated with alcohol liver disease, only a handful of cases have been reported on IgAV [8,9,10,15]. According to a case report published in December 2021, only 13 cases had previously reported the rare association between liver cirrhosis and HSP [15]. Another case study revealed how a patient with underlying hepatitis C consequently developed HSP. Hepatitis C is linked with mixed cryoglobulinemia, and thus altered metabolism of IgA immune complexes [16]. IgAV can eventually resolve on its own, without the use of steroids that are used in the case of nephropathy. Treating the underlying cause of cirrhosis will lead to the treatment of IgAV and nephropathy [8].

## Conclusions

Clinicians must be aware of elevated IgA levels and IgA immune complexes in adults with alcoholic cirrhosis. These IgA immune complexes can trigger immune-mediated vasculitis in these individuals. Our patient's rare presentation and the hunt involved in his diagnosis serves as a good refresher on the etiologies of small vessel vasculitides that clinicians should be aware of when evaluating purpuric rashes.
